# Bacterivorous Ciliate *Tetrahymena pyriformis* Facilitates *vanA* Antibiotic Resistance Gene Transfer in *Enterococcus faecalis*

**DOI:** 10.3390/antibiotics14050448

**Published:** 2025-04-28

**Authors:** Temilola O. Olanrewaju, James S. G. Dooley, Heather M. Coleman, Chris McGonigle, Joerg Arnscheidt

**Affiliations:** 1 School of Geography and Environmental Sciences, Ulster University, Cromore Road, Coleraine BT52 1SA, UKc.mcgonigle@ulster.ac.uk (C.M.); 2 Nutrition Innovation Centre for Food and Health (NICHE), School of Biomedical Sciences, Ulster University, Cromore Road, Coleraine BT52 1SA, UK; jsg.dooley@ulster.ac.uk; 3 School of Pharmacy and Pharmaceutical Sciences, Ulster University, Cromore Road, Coleraine BT52 1SA, UK

**Keywords:** antimicrobial resistance, antibiotic resistant bacteria, horizontal gene transfer, conjugation, protists, protozoa

## Abstract

**Background:** Wastewater treatment plants (WWTPs) are hotspots for the emergence and spread of antibiotic resistance genes (ARGs). In activated sludge treatment systems, bacterivorous protozoa play a crucial role in biological processes, yet their impact on the horizontal gene transfer in Gram-positive enteric bacteria remains largely unexplored. This study investigated whether the ciliate *Tetrahymena pyriformis* facilitates the transfer of antibiotic resistance genes between *Enterococcus faecalis* strains. **Methods**: Conjugation assays were conducted under laboratory conditions using a *vanA*-carrying donor and a rifampicin-resistant recipient at an initial bacterial concentration of 10^9^ CFU/mL and ciliate density of 10^5^ N/mL. **Results**: Transconjugant numbers peaked at 2 h when experiments started with recipient bacteria harvested in the exponential growth phase, and at 24 h when bacteria were in the stationary phase. In both cases, *vanA* gene transfer frequency was highest at 24 h (10^−4^–10^−5^ CFU/mL), and the presence of energy sources increased gene transfer frequency by one order of magnitude. **Conclusions**: These findings suggest that ciliate grazing may contribute to *vanA* gene transfer in WWTP effluents, potentially facilitating its dissemination among permissive bacteria. Given the ecological and public health risks associated with *vanA* gene persistence in wastewater systems, understanding protozoan-mediated gene transfer is crucial for mitigating the spread of antibiotic resistance in aquatic environments.

## 1. Introduction

Municipal sewage arriving at wastewater treatment plants (WWTPs) contain high loads of human waste, antibiotic residues, and mobile genetic elements (MGEs), reflecting the antibiotic resistance profile of the human population within their service area [1,2]. Wastewater treatment plants (WWTPs) have been considered likely hot spots and reservoirs for spreading antibiotic resistance into the environment [3,4,5]. While wastewater treatment plants are intended to treat wastewater, they can inadvertently create conditions that promote horizontal gene transfer, exacerbating the spread of antibiotic resistance [6].

WWTPs play a significant role in the dissemination and persistence of vancomycin-resistant enterococci (VRE) in wastewater, surface water, and downstream aquatic environments [7]. The persistence of vancomycin resistance genes and resistant bacteria in effluents has been linked to the enrichment of *vanA*-harbouring enterococci [8,9]. A cross-country surveillance study in Europe reported that, while the relative abundance of most clinically relevant ARGs declined after treatment, *vanA* genes were consistently detected in both influent and effluent samples across all investigated WWTPs [10]. Although antibiotic consumption patterns within each country influenced ARG prevalence in influents, other factors may have contributed to the enrichment of *vanA* genes [11].

Protozoa and bacteria play a vital role in biological wastewater treatment, shaping microbial community structure and driving nutrient cycling. Bacteria facilitate the mineralisation of organic compounds, while protozoa enhance this process by excreting nutrients that stimulate bacterial metabolism and proliferation [12,13,14]. In many modern wastewater treatment plants (WWTPs), low sewage loads, and extended retention times support a high diversity of protozoa, with ciliates comprising up to 70% of the protozoan population [15]. Through their grazing activity, ciliates help regulate bacterial populations and promote bioaggregate formation, ultimately enhancing effluent quality [13].

There is increasing evidence that the grazing activity of ciliates and other filter-feeding organisms influences the ecology and evolution of antibiotic resistance [16,17,18,19]. Studies have suggested that ciliate vesicles, produced during bacterial ingestion, serve as ecological microniches that support conjugative gene transfer [20,21,22,23]. However, most research on ciliate-facilitated conjugation has focused on Gram-negative (G-) bacteria, such as E. coli and *Klebsiella oxytoca*, providing key insights into conjugation rates and optimal experimental conditions for studying ciliate-mediated gene transfer [21,22].

While extensive research has explored ciliate-facilitated conjugation in G- bacteria, little is known about their role in promoting gene transfer in G+ bacteria. This is particularly relevant given that it has been argued that ciliates exhibit preferential grazing, favouring G- bacteria, but will also graze on G+ bacteria [24]. However, the role of their interactions with G+ bacteria in facilitating HGT remains largely unknown. Understanding whether ciliates contribute to ARG dissemination in G+ bacteria, such as *Enterococcus faecalis*, is crucial for evaluating their role in the spread of antibiotic resistance. Furthermore, any potential link between *vanA* gene persistence in WWTP effluents and ciliate grazing requires further investigation under controlled conditions.

This study investigates whether the free-swimming ciliate *Tetrahymena pyriformis* facilitates the horizontal transfer of the *vanA* gene between a plasmid-bearing donor and a recipient strain of *E. faecalis*. The findings provide the first direct evidence that ciliates can mediate vancomycin resistance transfer in G+ enteric bacteria during active filtration.

## 2. Results

### 2.1. Effect of Ciliate Viability on Gene Transfer

**Figure 1:**
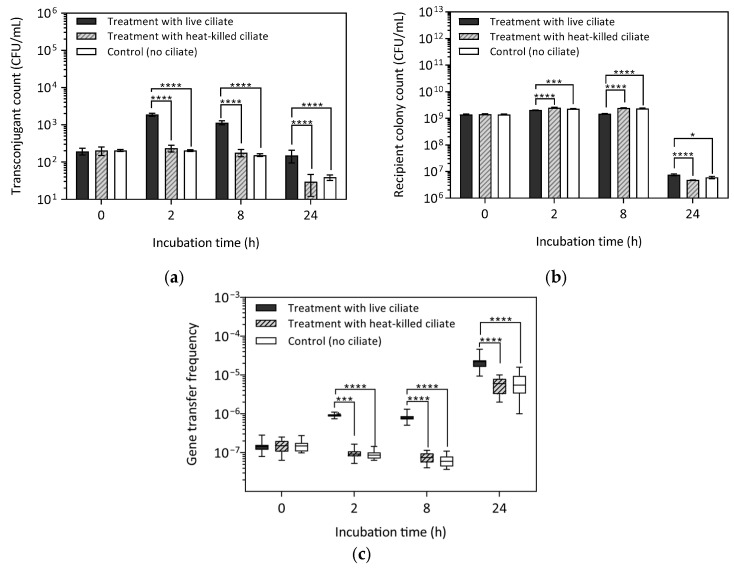
Conjugative *vanA* gene transfer between *E. faecalis* donor MF06036^Van^ and recipient ST02103^Rif^ harvested at 0, 2, 8, and 24 h with live and heat-killed ciliates. Data represent the standard error of the mean of 16 replicates. (**a**) Transconjugant count (**b**) Recipient colony count (**c**) Gene transfer frequency. The centre line in the box and whisker plot indicates the median transfer frequency. The box length represents the range of transfer frequency with the box edge at the 25th and 75th percentiles. Whiskers represent the minimum and maximum gene transfer frequency at each sampling interval. Asterisks indicate statistical significance: * *p* < 0.05, *** *p* < 0.001, **** *p* < 0.0001.

The effect of active ciliate grazing on gene transfer in *E. faecalis* was investigated using a donor strain (MF06036^Van^) and recipients (ST02103^Rif^), harvested in the mid-exponential phase. Conjugation assays were conducted with both live and heat-killed ciliates (Figure 1). It was hypothesised that horizontal gene transfer (HGT) via conjugation would result in the formation of transconjugants—recipient bacteria that acquired the *vanA* gene. To assess gene transfer, transconjugant count (TC), recipient colony count (RCC) and gene transfer frequency (GTF) were determined as the average of 16 replicates. Kruskal–Wallis (KW) test results indicated a significant difference in TC among the live-ciliate treatment, heat-killed ciliate, and control treatments at 2 h (H(2) = 32.8, *p* < 0.0001). Pairwise comparisons using Mann–Whitney U tests revealed that, at 2 h, TC was significantly higher in the live-ciliate treatment compared to both the killed (HK)-ciliate treatment (U(30) = 0, *p* < 0.0001) and the control (U(30) = 0, *p* < 0.0001; Figure 1a and Table S1). Similarly, at 8 h, TC remained significantly higher in the live-ciliate treatment than in the HK-ciliate treatments (U(30) = 0, *p* < 0.0001) and the control (U(30) = 0, *p* < 0.0001). However, compared to TC at 2 h, TC in the live-ciliate treatment at 8 h was significantly lower (U(30) = 0, *p* < 0.0001). At 24 h, a significant difference in TC was observed among the three groups (H(2) = 30.9, *p* < 0.0001), although TC was one order of magnitude lower than at 2 h across all groups. Additionally, no difference was found between the HK-ciliate treatments and the control group at 2 h U(30) = 81, *p* = 0.0774), 8 h (U(30) = 86.5, *p* = 0.1203), or 24 h (U(30) = 108.5, *p* = 0.4658). Similarly, TC in the HK-ciliate treatment and control was not significantly different (U(30) = 81, *p* = 0.0774). The presence of transconjugants in both the control and HK-ciliate treatments was not unexpected, as conjugation can occur through incidental contact between donor and recipient cells, including interactions with the walls of the Eppendorf tubes used in the assays.

Assessment of recipient colony count (RCC) showed that RCC was significantly lower in the live-ciliate treatment compared to both the HK-ciliate treatment and the control at 2 h (U (30) = 22.5, *p* < 0.0001; U(30) = 36, *p* < 0.00024) and at 8 h (U(30) = 0, *p* < 0.0001; U(30) = 0, *p* < 0.0001; Figure 1b; Table S1). However, at 24 h, RCC was significantly higher in the live-ciliate treatment than in both the HK-ciliate treatment (U(30) = 22, *p* < 0.0001) and the control (U(30) = 69, *p* = 0.0220). There was no difference between the HK-ciliate treatment and controls at 2 h (U(30) = 81.5, *p* = 0.0806), 8 h (U(30) = 112, *p* = 0.5563), or 24 h (U(30) = 92, *p* = 0.1132).

Gene transfer frequency (GTF) was determined as the ratio of transconjugant to recipient (Figure 1c and Table S1). At 2 h, the GTF in the live-ciliate treatment (9.2 × 10^−7^) was significantly higher than in the HK-ciliate (9.8 × 10^−8^; U(30) = 0, *p* < 0.0001) and the control (9 × 10^−8^; U(30) = 0, *p* < 0.0001), showing a one-order-of magnitude difference. A similar trend was observed at 8 h and 24 h, with GTF remaining highest in the live-ciliate treatment at 24 h. At this time point, the recipient population was lower than at 8 h in both control and treatment groups, leading to a higher proportion of transconjugants relative to total recipient colony count. Specifically, the recipient population exhibited a 2-log decrease (10^9^ to 10^7^) between 8 h and 24 h, while the transconjugant count declined by only 1-log decrease over the same period. This differential decline likely contributed to the elevated GTF at 24 h, suggesting that transconjugants made up a greater proportion of the recipient population at later time points.

**Figure 2:**
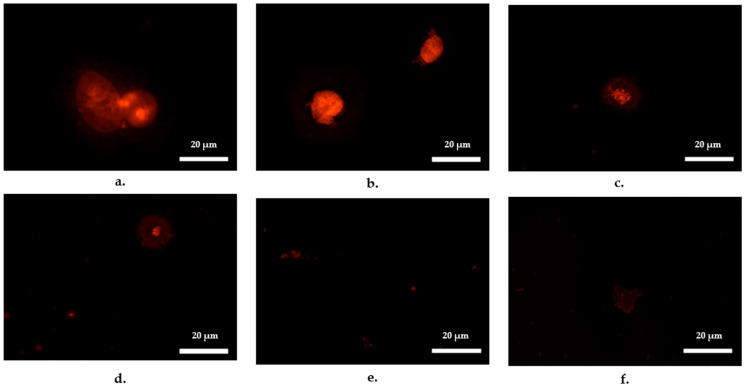
Images of fluorescence labelled *Enterococcus faecalis* inside *Tetrahymena pyriformis* after incubation periods of (**a**) 0.5 h (**b**) 1 h (**c**) 2 h (**d**) 4 h (**e**) 8 h and (**f**) 24 h in Page’s amoeba saline solution—ciliate vesicles with fluorescent *E. faecalis* are shown in the background (**d**–**e**). 10^9^ CFU/mL *E. faecalis* was incubated with 10^5^ N/mL *T. pyriformis* at 30 °C for 24 h. Fluorescence was viewed with a green excitation filter set at excitation/emission wavelength of 577/602 nm.

Fluorescence microscopy provided further insight into the early onset of conjugation, revealing bacterial accumulation within ciliate cells as early as 30 min into the conjugation assay (Figure 2). Within the first hour, fluorescent bacteria were visibly accumulating inside ciliates. By 2 h, bacterial cells had formed distinct aggregates within the ciliates, while vesicles containing bacterial cells began to appear in the background. At 4 h, bacterial aggregation within ciliates decreased, coinciding with an increased presence of bacteria-containing vesicles. By 8 h, vesicles carrying bacterial cells became more prominent in the background, and by 24 h, vesicles were still present, but at their lowest intensity observed throughout the experiment.

These findings suggest that active ciliates facilitated the conjugative transfer of the *vanA* gene between donor and recipient *E. faecalis* strains. The higher bacterial population observed in live-ciliate treatments compared to controls at 24 h (Figure 1b) suggests that bacterial cells may have derived energy or other advantages from ciliates, potentially enhancing their survival and proliferation.

### 2.2. Effect of Initial Bacterial Growth Phase on Gene Transfer

**Figure 3:**
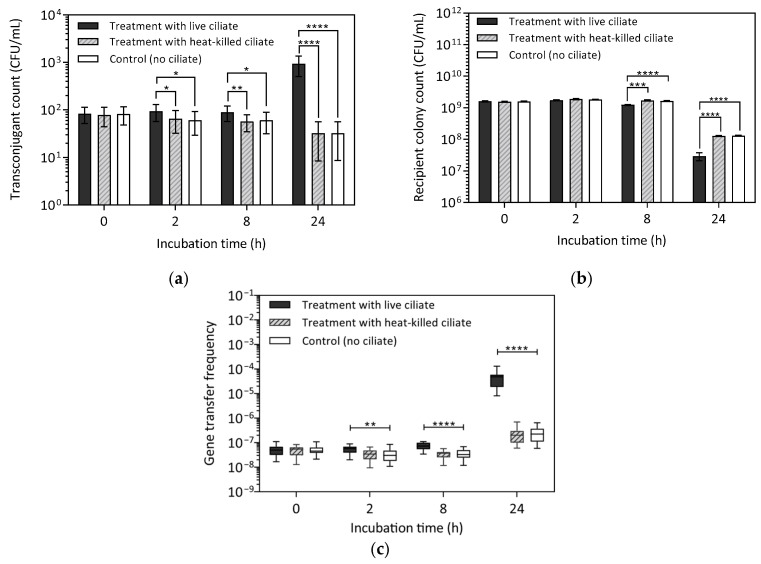
Conjugative *vanA* gene transfer between *E. faecalis* donor MF06036^Van^ and recipient ST02103^Rif^ harvested in the stationary growth phase (6 h) at 0, 2, 8, and 24 h with live and heat-killed ciliates. Data represent the standard error of the mean of sixteen replicates. (**a**) Transconjugant count (**b**) Recipient colony count (**c**) Gene transfer frequency. Asterisks indicate statistical significance: * *p* < 0.05, **p < 0.01, *** *p* < 0.001, **** *p* < 0.0001.

To assess the impact of the initial bacterial growth phase on gene transfer, conjugation assays were repeated using *E. faecalis* cells harvested in the stationary phase, referred to as stationary-at-inception assays. The assays involved treatments with live-ciliate treatments, heat-killed (HK) ciliate treatments, and controls without ciliates. Results showed transconjugant count (TC) was consistently higher in live-ciliate treatment compared to both the HK-ciliate treatment and the control across all sampling intervals (Figure 3a and Table S2). At 2 h, TC in the live-ciliate treatment was significantly higher than in the HK-ciliate treatments (U(30) = 66, *p* = 0.0178) and the control (U(30) = 65, *p* = 0.0160). A similar trend was observed at 8 h with TC in the live-ciliate treatment significantly higher than HK-ciliate treatment (U(30) = 55.5, *p* = 0.0051) and control (U(30) = 62.5, *p* = 0.0115). At 24 h, TC remained significantly higher in the live-ciliate treatment compared to the HK-ciliate treatment (U(30) = 0, *p* < 0.0001) and the control (U(30) = 4, *p* < 0.0001). There was no difference in TC between the HK-ciliate treatment and control at 2 h (U(30) = 120, *p* = 0.7719), 8 h (U(30) = 124.5, *p* = 0.9025), or 24 h (U(30) = 121.5, *p* = 0.8130).

When transconjugant count (TC) from the stationary-at-inception assays was compared to results from the exponential-at-inception assays where TC peaked at 2 h, a distinct trend emerged. In the live-ciliate treatment, TC reached its highest level at 24 h (938 ± 107 CFU/mL), which was one order of magnitude higher than TC at 2 h (94 ± 9 CFU/mL) and 8 h (89 ± 8 CFU/mL). TC at 2 h and 8 h in the live-ciliate treatment were not significantly different (U(30) = 119, *p* = 0.7507), suggesting that conjugation occurred at a low rate during the first 8 h and may have increased thereafter. When TC at 24 h in the stationary-at-inception assays was compared to exponential-at-inception assays, the closest TC value was observed at 8 h (1142 ± 35 CFU/mL). Notably, in the exponential-at-inception experiments, TC followed a downward trajectory from 2 h to 8 h, whereas in the stationary-at-inception assays, TC exhibited the opposite trend, increasing over time. These findings suggest that conjugation frequency at 24 h in the stationary-at-inception assays may be comparable to that at 8 h in the exponential-at-inception assays.

For recipient colony counts (RCC), no significant difference was observed between treatment and control groups at 2 h (H(2) = 3.331, *p* = 0.1891). However, at 8 h and 24 h, pairwise comparisons revealed significant differences in RCC between the live-ciliate treatment and the heat-killed (HK) ciliate treatment (8 h: U(30) = 34, *p* = 0.0002; 24 h: U(30) = 14.5, *p* < 0.0001) as well as between the live-ciliate treatment and the control (8 h: U(30) = 18.5, *p* < 0.0001; 24 h: U(30) = 14, *p* < 0.0001) (Figure 3b; Table S2).

When compared to the exponential-at-inception assays, RCC in the stationary-at-inception assays at 24 h (>2 × 10^7^ CFU/mL; Table S2) was more than twice the number in the exponential-at-inception assays (<1 × 10^7^ CFU/mL; Table S1). This higher recipient count suggests that ciliates exerted lower grazing pressure on stationary-phase bacteria at 24 h. Additionally, in control samples at 24 h, RCC declined from 10^9^ to 10^8^ CFU/mL in stationary-at-inception assays and from 10^9^ to 10^7^ CFU/mL in exponential-at-inception assays. This suggests a higher rate of cell death by 24 h when experiments were initiated with bacterial cells in the exponential growth phase.

Gene transfer frequency (GTF) was found to be significantly different between the treatment and control groups at all sampling intervals (2 h: H(2) = 9.543, *p* = 0.0085; 8 h: H(2) = 20.75, *p* < 0.0001; 24 h: H(2) = 31.45, *p* < 0.0001; Figure 3c; Table S2). However, no significant difference was observed between the heat-killed (HK) ciliate treatment and the control at 2 h (U(30) = 120, *p* = 0.7732), 8 h (U(30) = 122, *p* = 0.8305), or 24 h (U(30) = 118.5, *p* = 0.7304). Within the live-ciliate treatments, GTF varied significantly across time points, with the highest values recorded at 24 h. This elevated GTF was primarily driven by a decline in the recipient population, resulting in a higher transconjugant-to-recipient ratio compared to earlier time points.

### 2.3. Effect of Phenotypic Differences in Recipients on Gene Transfer

The temporal dynamics of *vanA* gene transfer in *E. faecalis* were investigated using a donor strain (MF06036^Van^) and two recipient strains (ST02103^Rif^ and MW01105^Rif^), harvested in the mid-exponential phase. Any residual transconjugants detected in treatments and controls at 0 h were most likely induced by centrifugation steps before incubation. After 2 h of incubation, TC was significantly higher in treatments than in controls (U(30) = 0, *p* < 0.0001). The highest transconjugant count (TC) was observed at 2 h for both recipient strains (Table 1 and Table S3). However, ST02103^Rif^ was more efficient in acquiring the *vanA* gene than MW01105^Rif^, with up to 50 times higher TC at 2 h and up to 100 times higher TC at 8 h and 24 h. In control samples, residual TC in ST02103^Rif^ treatments was approximately ten times higher than in MW01105^Rif^ samples between 0 h and 8 h, further highlighting a marked difference in pheromone-induced conjugation frequency between the two *E. faecalis* recipient strains.

When recipient colony count (RCC) was assessed, ciliate grazing pressure became evident by 2 h, as RCC was significantly lower in treatments compared to controls (U(30) = 49, *p* = 0.0021). By 24 h, RCC had decreased by more than two orders of magnitude, dropping to <10^7^ CFU/mL in treatments. However, there was no significant difference in RCC between treatments and controls (U(30) = 90.5, *p* = 0.1869). The similar RCC observed in both treatments and controls at 24 h suggests that, despite grazing pressure from ciliates, the recipient population in treatments was still maintained at levels comparable to controls.

### 2.4. Effect of Energy Source Availability on Gene Transfer

#### 2.4.1. Exposure of Bacteria to Spent Ciliate Culture Medium

**Figure 4:**
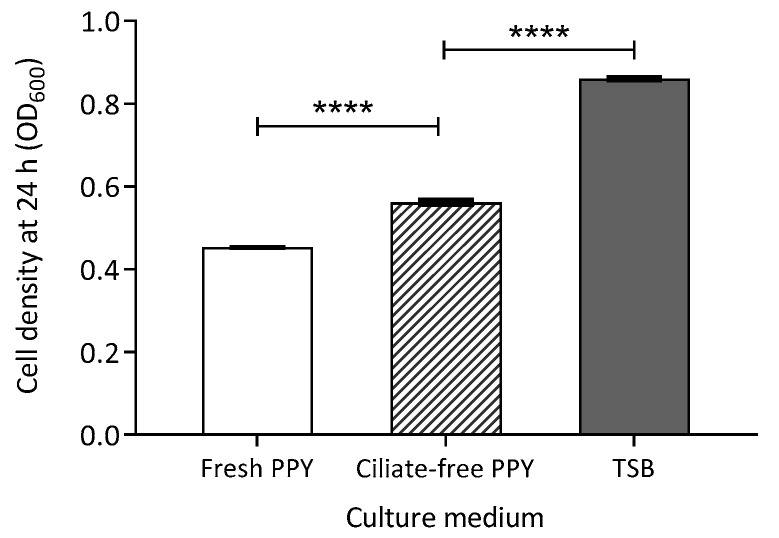
Optical density (600 nm) measurement of *E. faecalis* abundance in different culture medium after 24 h. Column = mean of nine replicates. Bar = standard error of the mean. Asterisks indicate statistical significance: **** *p* < 0.0001.

Bacterial cells were incubated in a proteose peptone yeast (PPY) medium, which had been previously used to culture ciliates but from which all ciliate cells had been removed. This medium is hereafter referred to as ciliate-free PPY or ciliate-conditioned medium. As a control, bacterial cells were also grown in tryptone soya broth (TSB) to obtain optimal growth readings, while fresh PPY served as a negative control. Optical density (OD) measurements, used as a proxy for recipient colony count (RCC), were significantly higher in ciliate-free PPY than in fresh PPY (Welch’s *t*-test: t(9) = 35.3, *p* < 0.0001). However, as expected, RCC in ciliate-free PPY was lower than in TSB (t(10) = 89.8, *p* < 0.0001; Figure 4 and Table S4). The increase in bacterial abundance suggests that *E. faecalis* utilised available nutrients in ciliate-free PPY. The observed difference between ciliate-free and fresh PPY may be attributed to the accumulation of organic waste from ciliates in the ciliate-conditioned medium, providing an additional energy source for bacterial growth.

#### 2.4.2. Exposure of Bacteria to Glucose as an Energy Source

**Figure 5:**
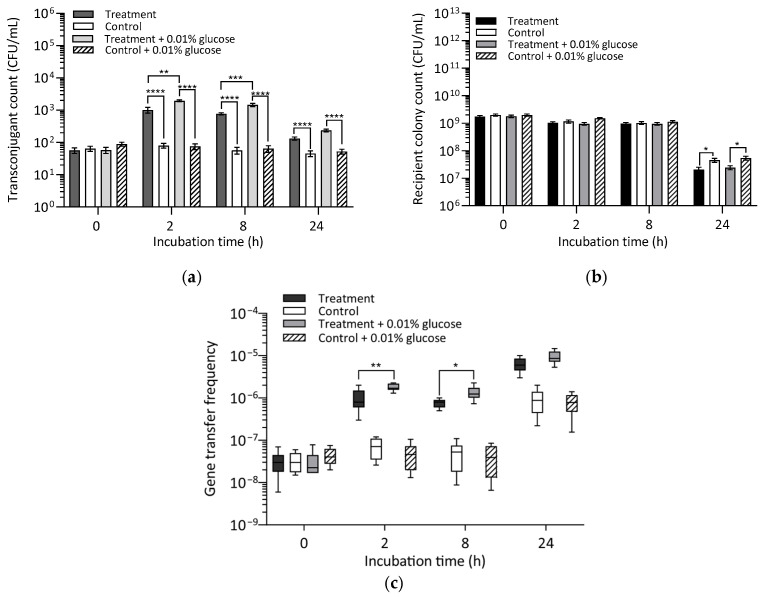
Effect of glucose availability on conjugative *vanA* gene transfer between *E. faecalis* donor MF06036^Van^ and recipient ST02103^Rif^ at 0 h, 2 h, 8 h and 24 h post incubation. Data represent the standard error of the mean of nine replicates. (**a**) Transconjugant count. (**b**) Recipient colony count. (**c**) Gene transfer frequency. Asterisks indicate statistical significance: * *p* < 0.05, **p < 0.01, *** *p* < 0.001, **** *p* < 0.0001.

The effect of an energy source on gene transfer frequency was further assessed by spiking conjugation treatments with 0.01% final glucose concentration (*w*/*v*) as an organic carbon enrichment to support additional bacteria growth. Assessment of transconjugant count (TC) showed that TC was significantly higher in treatments, both with or without glucose, compared to controls (U(16) = 0, *p* < 0.0001). Similar results were obtained at 8 h and 24 h (Figure 5a).

At 2 h, TC was significantly higher in glucose-spiked treatments compared to glucose-free treatments (U(16) = 8, *p* = 0.0028), a trend that persisted at 8 h (U(16) = 4.5, *p* = 0.0005) and 24 h (U(16) = 9, *p* = 0.0037). While TC decreased by one order of magnitude between 2 h and 24 h in both glucose-spiked and glucose-free treatments, TC in glucose-spiked treatments at 2 h (1943 ± 122 CFU/mL) was approximately twice as high as that of glucose-free treatments (1017 ± 204 CFU/mL). A similar twofold difference in TC was observed at 24 h (Table S5).

When recipient colony count (RCC) was assessed, RCC in controls was significantly higher than in both glucose-free (U(16) = 12.5, *p* = 0.0123) and glucose-spiked (U(16) = 12, *p* = 0.0106) ciliate treatments after 24 h (Figure 5b; Table S5). However, no significant differences in RCC were observed at 2 h or 8 h. Additionally, RCC did not significantly differ between glucose-spiked and glucose-free ciliate treatments at 2 h (U(16) = 34.5, *p* = 0.6171), 8 h (U(16) = 37, *p* = 0.7795), or 24 h (U(16) = 33, *p* = 0.5802). Likewise, no significant differences were detected between the control groups at 2 h (U(16) = 19.5, *p* = 0.0641), 8 h (U(16) = 33, *p* = 0.5309), or 24 h (U(16) = 32, *p* = 0.4730).

Gene transfer frequency (GTF) at 2 h and 24 h was twice as high in ciliate treatments (both glucose-spiked and glucose-free) compared to controls (Figure 5c and Table S5). The addition of glucose to ciliate treatments significantly enhanced gene transfer, as GTF was higher in glucose-spiked treatments than in glucose-free treatments at 2 h (U(16) = 8, *p* = 0.0028) and 8 h (U(16) = 14, *p* = 0.0185). However, at 24 h, no significant difference was observed between glucose-spiked and glucose-free treatments (U(16) = 28.5, *p* = 0.3071), suggesting that the additional energy source may have been depleted by this time, resulting in comparable GTF levels. These findings indicate that the presence of an energy source significantly influenced transconjugant formation only in the presence of ciliates, reinforcing the importance of a filter in facilitating conjugal contact between donor and recipient cells. In such cases, the availability of an energy source may support bacterial metabolic activity, promoting more efficient conjugation.

## 3. Discussion

Wastewater treatment plants are a highly suitable environment for gene transfer as they host a diverse mix of bacteria, antimicrobial agents, and nutrients [25,26,27]. In biological wastewater treatment systems, bacterivorous ciliates play a prominent role in grazing on dispersed bacteria, reducing bacterial biomass, and decreasing biological oxygen demand (BOD) of wastewater effluent [28]. *E. faecalis* can acquire resistance genes (ARGs) in municipal sewage treatment plants under natural conditions [29]. This study investigated the effect of ciliate grazing on the spread of the *vanA* genes in *E. faecalis*. It was hypothesised that horizontal gene transfer (HGT) between donor and recipient strains of *E. faecalis* within ciliate vesicles could contribute to the increase and persistence of the *vanA* gene through the formation of *vanA* gene-carrying transconjugants.

### 3.1. Effect of Ciliate Viability on Transconjugant Formation

*Tetrahymena pyriformis* engulfs planktonic bacteria, digesting them within food vacuoles in sequential bursts [30]. Previous studies on Gram-negative bacteria have demonstrated that conjugative transfer of ARGs occurs during engulfment in food vacuoles, facilitating horizontal gene transfer [21,23]. Given this, the present study investigated whether similar mechanisms contribute to gene transfer in *E. faecalis* by comparing conjugation in cocultures with live and heat-killed ciliates.

Transconjugant counts were higher in live-ciliate treatments than in heat-killed ciliate treatments or ciliate-free controls. Notably, transconjugant numbers in heat-killed ciliate treatments were not different from those in ciliate-free controls, indicating that active ciliate grazing plays a crucial role in facilitating conjugation. The early formation of transconjugants within the first 2 h, followed by a decline, is consistent with previous findings for E. coli [21,22]. By 24 h, *E. faecalis* transconjugant counts in controls remained near baseline levels (comparable to 0 h), whereas transconjugant numbers in live-ciliate treatments were one order of magnitude higher. These findings suggest that conjugation in *E. faecalis* occurs at a significantly higher frequency in the presence of live ciliates, with the most substantial transconjugant recovery observed within the first 2 h of grazing activity.

### 3.2. Effect of Bacterial Growth Phase on Transconjugant Formation

It was hypothesised that the high transconjugant numbers observed at 2 h in ciliate treatments were influenced by the growth phase of *E. faecalis*. To test this, we conducted conjugation experiments with *E. faecalis* in the stationary phase (hereafter referred to as stationary-at-inception) and compared the results to those obtained from exponential-phase cells (hereafter referred to as exponential-at-inception) under nutrient-limiting conditions. Between 2 h and 8 h, transconjugant numbers in exponential-at-inception assays were 20 times higher than those in stationary-at-inception assays. This suggests that ciliates exerted greater grazing pressure on exponential-phase *E. faecalis*, likely due to its higher metabolic activity. These findings align with previous studies on E. coli, where transconjugant counts peaked within 2 h, corresponding with the ciliates’ rapid engulfment of bacteria [21,22]. The 2 h mark also coincides with the digestion cycle of *Tetrahymena* sp. [30], after which vesicles containing intact or partially digested bacteria are egested [31]. While the bacterial growth phase was not explicitly stated in those studies, it can be inferred that metabolically active *E. coli* cells were used, given the similarity in gene transfer frequencies between those studies and our findings on enterococci.

Since higher metabolic activity favours gene transfer [32], it is likely that the metabolic state of the bacterial cells during the first 8 h influenced both ciliate grazing pressure and conjugation efficiency. Notably, gene transfer frequency remained significantly higher in stationary-phase cells exposed to live ciliates than in those exposed to heat-killed ciliates and controls, suggesting some level of grazing by the ciliates. However, transconjugant numbers in stationary-at-inception assays remained low between 2 h and 8 h, indicating either reduced metabolic activity by the stationary bacteria or reduced grazing by the ciliates during this period. It has been argued that ciliates exhibit preferential grazing, favouring larger, metabolically active bacterioplankton [33]. Studies have reported that stationary-phase *E. faecalis* can persist in protozoan-rich environments, with up to 100% recovery of intact stationary-phase cells after a 7-day incubation, whereas exponential-phase *E. faecalis* became undetectable after 4 days [34]. After being egested from ciliate vesicles, stationary-phase cells may have undergone morphological adaptations and developed stress resistance that supported their survival [35].

In stationary-at-inception assays, bacterial abundance remained stable during the first 2 h, whereas in exponential-at-inception assays, bacterial abundance in ciliate treatments was significantly lower than in controls, indicating higher grazing pressure. This aligns with previous reports showing that *E. faecalis* in the exponential growth phase is more readily digested than in the stationary phase [34]. In this study, the impact of ciliate grazing on *E. faecalis* abundance in stationary-at-inception assays became evident at 24 h, coinciding with a one-order-of-magnitude increase in transconjugant count compared to ciliate-free controls. At this time point, transconjugant count in stationary-at-inception assays was ten times higher than in exponential-at-inception assays, and the recipient cell abundance in stationary-at-inception assays was also one order of magnitude higher than in exponential-at-inception assays.

A shift in transconjugant trends was observed over time. At 2 h, transconjugant counts in stationary-at-inception assays were 100-fold lower than in exponential-at-inception assays. However, after 24 h, the trend reversed, with stationary-at-inception assays producing ten times more transconjugants than exponential-at-inception assays. This suggests that *E. faecalis* in the stationary phase may have re-entered the exponential growth after 8 h, leading to increased bacterial abundance and enhanced ciliate grazing pressure. Since enterococci are capable of transitioning between growth phases depending on nutrient availability [36], a possible explanation for this shift is the accumulation of ciliate-derived organic waste in stationary-at-inception assays, which may have provided additional nutrients that stimulated bacterial metabolism and growth.

### 3.3. Effect of Distinct Recipient Phenotypes on Gene Transfer Frequency

Gene transfer frequency in ciliates was assessed over a 24 h period in conjugation assays to evaluate transconjugant formation dynamics. Recipient strains with varying conjugation efficiencies (MW01105^Rif^ and ST02103^Rif^) were selected based on the premise that gene transfer may occur at different rates in dynamic systems such as wastewater treatment plants (WWTPs). For the exponentially growing recipient strain MW01105^Rif^, transconjugant counts (TC) in ciliate treatments were significantly higher than in ciliate-free controls. Maximum transconjugant numbers were achieved within 2 h, followed by a decline over the next 24 h. This trend aligns with previous studies on E. coli, where transconjugant counts peaked within 2 h of incubation [21,22]. Additionally, a greater reduction in recipient counts was observed in ciliate treatments compared to ciliate-free controls, suggesting that protozoan grazing influences recipient survival dynamics. A similar pattern was observed with the second recipient strain, ST02103^Rif^, though transconjugant counts in ciliate treatments were consistently two orders of magnitude higher than those of MW01105^Rif^. This result reinforces previous studies that highlighted differences in the conjugation efficiency of these two *E. faecalis* recipient strains [18]. The rapid conjugation events observed early in the experiment likely resulted from initial ciliate ingestion of planktonic bacteria, as ciliates have demonstrated a greater capacity for clearing high-density bacterial populations than lower-density populations [30].

A common trend across experiments was that gene transfer frequency was consistently one order of magnitude higher in ciliate treatments than in ciliate-free controls. This result aligns with other studies where an order-of-magnitude increase in kanamycin resistance gene transfer was observed in ciliate-treated *E. coli* strains compared to ciliate-free controls [21]. In other studies, ciliate presence has been shown to increase E. coli gene transfer by up to three orders of magnitude [20]. Differences in observed conjugation frequencies may be influenced by the type of resistance gene selected [37], the conjugation efficiency of recipient strains, or structural differences between Gram-positive and Gram-negative bacteria [38].

Protozoan grazing studies suggest that ciliates preferentially digest Gram-negative bacteria over Gram-positive bacteria [38]. The decline rate of faecal coliforms in the presence of protozoa is reported to be three to five orders of magnitude higher than for faecal streptococci [39]. This discrepancy has been attributed to differences in susceptibility to protozoan grazing and variable digestion rates within ciliate vesicles [38]. Compared to Gram-negative rods, Gram-positive cocci appear to be digested at a substantially lower rate [33,40]. The complexity of the Gram-positive cell wall may play a role in slowing enzymatic digestion within ciliate vesicles [31,38]. This prolonged retention of *E. faecalis* within ciliate vesicles may facilitate gene exchange before bacterial egestion, potentially contributing to the release of viable transconjugants.

### 3.4. Effect of Energy Source Availability on Transconjugant Formation

The increase in recipient colony counts observed in live-ciliate treatments at 24 h in exponential-at-inception experiments suggests that ciliate-derived metabolic byproducts may enhance bacterial proliferation, potentially by supplying accessible nutrients. To investigate this, *E. faecalis* was grown in a ciliate-conditioned medium and compared with growth in fresh PPY medium (control) and standard bacterial culture medium (tryptone soya broth). Optical density (OD600) was used as a proxy for bacterial abundance. The results showed that *E. faecalis* growth in the ciliate-conditioned medium was significantly higher than in the unconditioned fresh PPY control medium, suggesting that metabolites released by ciliates may support bacterial proliferation. However, OD600 values remained approximately 50% lower than those observed in standard bacterial culture medium. The accumulation of growth-stimulating compounds excreted by protozoa may account for the increased bacterial abundance in the ciliate-conditioned medium [41,42]. Protozoa are known to excrete organic nutrients, such as amino acids, which contribute to dissolved organic carbon and influence bacterial metabolism and growth [43,44].

Variations in transconjugant and recipient colony counts between exponential-at-inception and stationary-at-inception assays suggest that ciliate organic waste may accumulate when grazing pressure decreases, potentially influencing bacterial growth. The higher recipient colony counts observed in stationary-at-inception assays support this assumption. *Tetrahymena* sp. are known to excrete capsular mucous materials rich in carbohydrates and nucleic acids, which facilitate bio-aggregate formation and microbial interactions during wastewater treatment [45,46].

To further investigate the effect of energy resources on conjugation frequency, conjugation assays were supplemented with 0.01% glucose, corresponding to a 100 mg/L BOD [12]. The addition of glucose doubled the number of transconjugants in ciliate treatments, indicating that glucose availability supplemented the energy demand for bacterial metabolism [47]. Increased energy availability also sustained bacterial abundance, as evidenced by higher bacterial biomass in glucose-spiked controls compared to glucose-free controls. The presence of both ciliates and an additional carbon source appears to have a synergistic effect on conjugation frequency. Protozoa contribute to bacterial metabolism in wastewater treatment plants (WWTPs) by excreting mineral nutrients that enhance carbon utilisation [14,47]. Given that high organic content is a characteristic of WWTP influent compartments, where both ciliates and bacteria are abundant [13,48,49], these findings suggest that high metabolic activity within ciliate vesicles may contribute to elevated gene transfer frequencies in *E. faecalis*, particularly in secondary treatment processes.

### 3.5. Limitations of Conjugation Studies

#### 3.5.1. Ciliate Grazing and Transconjugant Formation

This study did not measure the rate at which ciliates grazed on *E. faecalis*, despite their ability to consume both Gram-negative and Gram-positive bacteria. While fluorescence imaging confirmed *E. faecalis* ingestion by ciliates, the exact grazing rate was not quantified. Additionally, experiments were conducted at bacterial concentrations optimised for conjugation studies in ciliate-bacteria cocultures, which may not fully represent bacterial densities typically found in wastewater treatment plants.

Fluorescence staining of *E. faecalis* was used to confirm the presence of bacteria within ciliates, but this method did not directly confirm transconjugant formation inside vesicles. Real-time detection of conjugation events would require fluorescence-tagged donor and recipient strains to monitor conjugative transfer. However, applying such techniques to environmental enterococci isolates carrying the *vanA* gene presents methodological challenges, necessitating the use of fluorescence staining as a proxy for tracking bacterial aggregation within ciliates.

Gene transfer frequency within the entire conjugation system ranged between 10^−7^ and 10^−5^, as measurements were based on the whole supernatant, which included ciliates, vesicles, and extracellular bacteria. However, the exact frequency of conjugation occurring specifically within ciliate vesicles was not determined in this study. Isolating and purifying ciliate vesicles for selective plate counts would be necessary to quantify intracellular conjugation events [21]. Despite this limitation, the findings remain valid, as transconjugants formed both within and outside vesicles were accounted for in the total system. Further studies isolating ciliate vesicles would provide a clearer understanding of their role in conjugation.

#### 3.5.2. Use of Selective Media in Transconjugant Detection

Culture-dependent methods have been widely used for isolating and quantifying specific microorganisms [50]. Multidrug-resistant (MDR) *Enterococcus* spp. has been detected in approximately 29% of treated wastewater effluent using culture-dependent selective media [51]. In this study, selective media containing defined concentrations of vancomycin and rifampicin were used to isolate transconjugants that had acquired vancomycin resistance during conjugation studies. However, selective plating may underestimate transconjugant abundance, as viable but non-culturable (VBNC) enterococci with newly acquired *vanA* genes may be present but undetectable in standard culture conditions [52].

The slow digestion rate of *E. faecalis* within ciliate vesicles [53] suggests that a significant proportion of enterococci egested after the initial 2 h digestion cycle may exist in a VBNC state [54]. As a result, transconjugant numbers detected using selective media may be underestimated, as not all viable transconjugants would have formed visible colonies on agar plates. To improve accuracy, quantitative PCR (qPCR) can be used to quantify ARGs, while recombinase polymerase amplification (RPA) assays, which have demonstrated 100% specificity for *E. faecalis* in wastewater, offer a promising alternative for enhanced detection [55,56]. Ultimately, standardised recovery methods for antibiotic-resistant pathogens are essential for improving surveillance and risk assessment in wastewater treatment plants [57].

#### 3.5.3. Ciliate Waste Products as Potential Energy Sources

While the preliminary findings in Section 3.4 suggest that ciliate-conditioned media support bacterial survival, they do not establish a direct metabolic enhancement or a causal link between bacterial abundance and conjugation efficiency. Although a larger recipient population could theoretically increase conjugation potential, OD_600nm_ readings alone do not confirm whether ciliate waste directly influenced bacterial growth or transconjugant formation. Further research is needed to determine whether ciliate-derived metabolic byproducts sustain bacterial populations and enhance conjugation frequency in nutrient-limited environments. This would require identifying and quantifying specific components of ciliate-conditioned PPY medium to evaluate their role in bacterial metabolism.

#### 3.5.4. Bacterial Growth Phase and Gene Transfer Frequency

Variations in transconjugant counts between experiments initiated with exponential-phase versus stationary-phase bacteria suggest that the bacterial growth phase influences conjugation efficiency. Given that the exponential phase is critical for gene transfer, the higher transconjugant count observed at 24 h compared to earlier time points in stationary-at-inception experiments may indicate that enterococci cells had transitioned into a rapid growth phase. This shift may have coincided with intensified ciliate grazing, supported by lower recipient colony counts in live-ciliate treatments compared to controls.

The observed increase in transconjugants at 24 h suggests that stationary-phase bacteria may have re-entered active growth, potentially facilitating higher conjugation rates. This aligns with previous studies showing that bacterial conjugation is more efficient in actively dividing populations. However, further investigation is needed to elucidate the role of growth-phase transitions in conjugation assays and to explore potential links between ciliate waste accumulation and bacterial proliferation.

### 3.6. Implications for Wastewater Treatment Plants

Plasmids play a central role in horizontal gene transfer (HGT) among prokaryotes, enabling the exchange of accessory genes that enhance bacterial adaptation and survival [58]. In *E. faecalis*, conjugation occurs through pheromone-responsive plasmid systems, such as pCF10, which was used in this study [59,60]. The transfer of conjugative plasmids is a key factor in the persistence of antibiotic resistance genes (ARGs) within ciliate-bacteria systems, as bacteria with defective conjugation mechanisms may be more susceptible to ciliate predation [61]. Beyond facilitating conjugation within vesicles, ciliate grazing may also release genetic material from partially digested bacteria into the environment [62]. This accumulation of extracellular ARGs provides a reservoir for transformation-based HGT, contributing to the persistence of ARGs in wastewater effluents, even when host bacterial abundance is low [63]. Once released, these extracellular ARGs persist in WWTP influents, where they may be taken up by permissive bacteria and further propagated across treatment compartments [64]. ARG abundance in effluents may increase through selective enrichment or additional conjugation events, reinforcing the role of protozoa in facilitating *vanA* transfer in WWTPs. The emergence of permissive bacteria in the environment poses a significant public health risk, given the immense potential for ARG dissemination and exchange with human pathogens [65,66].

The persistence of *vanA* in wastewater effluents is likely driven by complex interactions between protozoa, permissive bacteria, and broad-host-range plasmids. A study in Germany found that enterococci abundance declined in WWTP effluents, while *vanA* gene abundance increased by several orders of magnitude, indicating continued gene propagation despite bacterial reductions [67]. Similarly, another study reported a 99% reduction in enterococci, yet *vanA* gene abundance increased significantly [68]. These findings suggest active HGT among closely related and phylogenetically diverse bacterial populations within wastewater compartments.

Within WWTPs, plasmid shuttles—bacterial groups that acquire and transfer conjugative plasmids—play a crucial role in ARG dissemination. These bacteria facilitate HGT in activated sludge and biofilms, where high bacterial densities enhance plasmid exchange. Permissive bacteria, including species from the Enterobacteriaceae, Pseudomonadaceae, and Acinetobacter lineages, act as major transconjugants, ensuring the persistence and spread of ARG-bearing plasmids across treatment stages [64].

Among these, *Arcobacter* spp. have been identified as potential keystone bacteria in wastewater environments [69,70]. They are widespread in animal hosts and the environment [67]. Recognised as emerging pathogens with zoonotic potential [70], the genus *Arcobacter* has been associated with HGT in WWTPs [71] and human infections through contaminated dairy products [67,72]. Like *Enterococcus*, *Arcobacter* spp. are abundant in wastewater [69] and carry ARG plasmids that confer resistance across diverse hosts [64]. One study reported that *Enterococcus* and *Arcobacter* were prevalent in hospital wastewater, each accounting for multiple ARG types, with some instances representing 50% of detected ARGs [73]. The widespread detection of *vanA* genes in wastewater highlights the role of bacteria harbouring broad-host-range plasmids in maintaining ARGs despite standard treatment processes, potentially facilitating their dissemination into environmental reservoirs. However, no direct link has been established between *Arcobacter* and *vanA* gene abundance in wastewater.

Although WWTPs serve as critical control points for ARG mitigation, conventional treatment processes alone are often insufficient in eliminating resistance genes. Studies have shown that *vanA* gene abundance can increase within surviving bacterial populations, even after advanced treatment interventions such as ozone disinfection [64]. Given that traditional WWTPs were not originally designed to remove ARGs [73], a multi-stage treatment approach—integrating membrane filtration, advanced oxidation processes, and bioaugmentation—may be necessary to achieve effective ARG reduction in wastewater effluents [74]. Further research is required to optimise these treatment strategies, ensuring greater efficiency in ARG removal and mitigation of antibiotic resistance spread [75].

## 4. Materials and Methods

### 4.1. Protozoa

*Tetrahymena* spp. are free-living ciliated protozoans found in freshwater lakes and ponds. They are typically pear to ovoid-shaped with a size range of 30–50 µm [76]. Axenic *T. pyriformis* strains were acquired from the Culture Collection of Algae and Protozoa (1630/1W; CCAP, Oban, Scotland). Ciliates were statically cultured in proteose peptone yeast (PPY) extract media (Composition per litre: Proteose peptone: 20 g (Oxoid L85, Oxoid, Basingstoke, England); yeast extract: 2.5 g (Oxoid L21, Oxoid, Basingstoke, England) in universal tubes and incubated at 10 °C. For rapid growth, cultures were subcultured in 12 mL volumes of PPY and incubated at 20 °C under low lighting on a 12 h L: 12 h D cycle. *T. pyriformis* cultures were grown to a cell concentration of approximately 10^5^ N/mL for 7 days. Ciliates were collected by centrifugation (300× *g*, 3 min, 20 °C) and resuspended in Prescott’s and James’ (PJ) solution (Stock composition per 100 mL: (1) 0.43 g CaCl_2_.2H_2_0, 0.16 g KCl; (2) 0.51 g K_2_HPO_4_ (3) 0.28 g MgSO_4_. 7H_2_O; 1 mL of stocks 1–3 made up to 1000 mL with deionised water and autoclaved at 121 °C for 15 min). Ciliates were incubated at 20 °C overnight in PJ solution without feeding to increase the rate of food uptake during the experiment. The ciliate population was determined using a Sedgewick Rafter counting chamber (Pyser Optics, Edenbridge, UK).

### 4.2. Bacteria

*Enterococcus faecalis* strains used in this study have been previously described [18]. The investigation of conjugative gene transfer involved an *E. faecalis* donor strain, MF06036^Van^, carrying a vancomycin resistance gene-bearing plasmid, and two recipient strains, MW01105^Rif^ or ST02103^Rif^, resistant to rifampicin. The recipients were selected based on their ability to acquire and retain a conjugative plasmid carrying the *vanA* gene, and their differing conjugation efficiencies, which reflect the natural strain variation seen within *E. faecalis* conjugation systems. The antimicrobial resistance profile of the strains has also been previously described [18]. Static overnight bacterial cultures were grown in Tryptone Soya Broth (TSB, Oxoid CM0129, Basingstoke, England) at 37 °C, streaked onto Tryptone Soya Agar (TSA, Oxoid, CM0131, Basingstoke, England) and maintained at 4 °C for the entire duration of the study.

#### Preparation of Fluorescence-Stained *Enterococcus faecalis*

Preparation of a stock solution of red fluorescence dye, Cell Tracker Red CMTPX (Thermo Fisher Scientific Inc., Waltham, MA, USA), involved warming a dye vial containing 50 µg of red fluorescence dye from its −20 °C storage temperature to room temperature and dissolving the dye in approximately 7 µL of Dimethyl Sulfoxide (DMSO) to achieve a 10 mM concentration. A working solution of 25 µM concentration was prepared by adding 5 µL of stock solution to 2 mL of cell-free spent *E. faecalis* broth culture and incubating at 37 °C. A 5 mL of 90 min *E. faecalis* culture grown to a density of 10^9^ CFU/mL was diluted to 10^8^ CFU/mL in tryptone soya broth and centrifuged at 2000× *g* for 10 min, after which the supernatant was discarded. The bacterial pellet was resuspended in 2 mL of the prewarmed working dye solution and incubated at 37 °C for 45 min. After another centrifugation step, the supernatant was discarded, and the cell pellets were washed four times in PBS before resuspension in TSB.

### 4.3. Reagents

Vancomycin and rifampicin were purchased from Sigma-Aldrich (St. Louis, MO, USA). Final concentrations of 10 µg/mL vancomycin and 100 µg/mL rifampicin were used for transconjugant selection. Cycloheximide, for the inhibition of protein synthesis, was purchased from EMD Millipore Corp (Burlington, MA, USA). Latrunculin B, an actin polymerisation inhibitor, was acquired from Sigma-Aldrich (St. Louis, MO, USA).

### 4.4. Conjugation Studies

#### 4.4.1. Conjugation Assay—Effect of Ciliate Viability

Conjugation experiments were conducted to assess the frequency of antimicrobial resistance gene transfer between *E. faecalis* strains in the presence of live (viable) ciliates, using previously described methods [21]. Equal concentrations of 10^9^ CFU/mL *E. faecalis* donor strain MF06036^Van^ and recipient strain ST02103^Rif^ were cultured in TSB at 37 °C for 90 min to mid-exponential growth phase. Bacterial cultures were individually centrifuged at 2500× *g* for 10 min. Supernatants were discarded, and pellets were washed by resuspension and centrifugation in Page’s amoeba saline (PAS; Composition per litre: KH_2_PO_4_ 0.136 g, Na_2_HPO_4_ 0.142 g, MgSO_4_.H_2_O, NaCl 0.12 g, CaCl_2_.6H_2_O 0.4 mg, pH 6.8; Page, 1988). Bacterial concentrations in the conjugation treatment were adjusted according to optical density before incubation. At the same time, ciliate abundance was determined using the trypan blue dye exclusion method (Trypan blue solution from Sigma, St. Louis, MO, USA). A 10^5^ N/mL ciliate culture was centrifuged at 250× *g* for 3 min before resuspension in PAS.

Conjugation treatment samples were prepared by mixing 0.5 mL each of donor and recipient *E. faecalis* with 1 mL ciliate culture containing 10^5^ N/mL ciliate in 2.5 mL Eppendorf tubes, and conjugation treatments and controls (without ciliates) were then incubated at 30 °C for 24 h. Transconjugant counts were determined at 0 h, 2 h, 8 h, and 24 h. For this, 100 µL of samples were transferred to 1.5 mL Eppendorf tubes, subjected to bead-beating following [21], and then plated onto selective TSA. Transconjugants were isolated on selective TSA containing 10 μg/mL vancomycin and 100 μg/mL rifampicin. At each sampling time, recipient concentrations were also determined by selection on TSA plates containing only 100 μg/mL rifampicin. Gene transfer frequency was defined as the ratio of transconjugants to recipient colony count. Experiments were performed using live ciliates and heat-killed ciliates to confirm that antimicrobial resistance gene transfer between *E. faecalis* strains occurred through active grazing by ciliates and not just by attachment to ciliate surfaces. Heat-killed ciliates were prepared by 10 min incubation in a water bath at 90 °C. Experiments were initially conducted with six replicates per treatment and repeated twice with five replicates each to make 16 replicates.

##### Emergence of Bacterial Cells Within Ciliates

To assess the accumulation of bacterial cells within ciliate vesicles, treatment samples with 10^5^ N/mL ciliate culture were incubated with 10^9^ CFU/mL of fluorescence-stained donor *E. faecalis* cells only following methods described under Section 4.4.1 at 30 °C for 4 h. Fluorescence-stained *E. faecalis* within ciliates were viewed with a 40× objective lens on a Nikon Eclipse E400 fitted with a Nikon DS-Fi1C using a G2-A (green excitation) filter set (excitation/emission wavelengths: 577/602 nm). Fluorescence images were captured with NIS-elements software (Version 4.50.x).

#### 4.4.2. Conjugation Assay—Effect of Phenotypically Distinct Recipients

Using the best-performing ciliate abundance, conjugation experiments were repeated with 10^9^ CFU/mL concentration of the *E. faecalis* donor strain MF06036^Van^ and two recipient strains, MW01105^Rif^ and ST02103^Rif^, grown to mid-exponential growth phase. Treatments and controls were incubated for 24 h and 100 µL of samples were collected for bead-beating and plating at 0, 2, 8, and 24 h incubation intervals, as described under Section 4.4.1. Experiments were conducted with 6 replicates and repeated twice with 5 replicates each to make 16 replicates.

#### 4.4.3. Conjugation Assay—Effect of Starting Bacterial Growth Phase

Conjugation experiments conducted in Section 4.4.1 with bacteria harvested in the mid-exponential growth phase were repeated with stationary phase *E. faecalis* following the same method to assess how the bacterial growth phase may affect gene transfer frequency. Overnight donor and recipient strains were grown for 6 h to the stationary growth phase and harvested for conjugation experiments. The experiment was performed in 16 replicates: an initial six replicates followed by two repeats of five replicates each.

#### 4.4.4. Conjugation Assay—Effect of Energy Source Availability

##### Exposure of Bacteria to Spent Ciliate Culture Medium

To determine the potential effect of ciliate waste on *E. faecalis* growth, 40 mL of a ciliate culture grown in PPY in a 50 mL centrifuge tube was centrifuged at 4500× *g* at 4 °C for 10 min. The supernatant was decanted into another 50 mL centrifuge tube. Centrifugation and decanting were repeated until no ciliate pellets were observed at the base of the tube. Light microscope inspection of a 1 mL supernatant sample (hereafter called ciliate-free spent PPY) in a Sedgewick Rafter counting chamber after a filtration step using a 0.2 µm sterile filter confirmed the complete removal of ciliates. A mid-exponential phase *E. faecalis* culture was prepared, and 9 mL of ciliate-free PPY was inoculated with 1 mL of bacterial culture to make a 10% treatment. As controls, 10% of bacterial cultures were prepared in fresh PPY and TSB. All treatments were incubated at 37 °C for 24 h. Optical density was determined at 600 nm using a UV/Vis spectrophotometer (UV-1800, Shimadzu Corp., Kyoto, Japan).

##### Exposure of Bacteria to Glucose as an Energy Source

The effect of energy availability on conjugative gene transfer was further tested by spiking ciliate conjugation treatments with a 0.01% *w*/*v* final glucose concentration. Ciliate conjugation treatments and controls without glucose served as negative controls. All experiments were conducted three times and, each time, in triplicate.

### 4.5. Statistical Analysis

Statistical analysis was conducted using the GraphPad Prism software (Version 8.0). To determine the effect size, a power analysis was performed using G*Power (Version 3.1), based on preliminary data from a pilot experiment with nine replicates per group. The analysis indicated that four replicates per group were sufficient to detect a 20% difference in the median at α = 0.05 and power = 0.9, using the Mann–Whitney U test. To enhance the robustness of the study, 16 replicates per group were used in the final conjugation experiments. Due to the small sample size, non-parametric tests were chosen for statistical analysis. The Mann–Whitney U test was used for pairwise comparisons between experimental treatments and controls, while the Kruskal–Wallis test was used to assess differences across multiple groups at a 5% significance level. For bacterial density measurements, OD_600_ readings were analysed using Welch’s *t*-test, following checks for normality and homogeneity of variances.

## 5. Conclusions

This study provides the first direct evidence that bacterivorous ciliates facilitate antibiotic resistance gene (ARG) transfer in Gram-positive bacteria, expanding previous findings in Gram-negative species. It was hypothesised that ciliate grazing facilitates the dissemination of vancomycin resistance genes among enterococci. Results showed that *Tetrahymena pyriformis* induced the formation of transconjugant *E. faecalis* that acquired the *vanA* gene during a 2–24 h experimental period. Transconjugant formation peaked at 2 h in assays initiated with *E. faecalis* harvested in the exponential growth phase, aligning with findings on conjugation in Gram-negative enteric bacteria within ciliate vesicles. The physiological state of enterococci influenced transconjugant abundance, with assays initiated in exponential and stationary growth phases producing distinct transconjugant counts over time. The availability of energy sources also influenced gene transfer, with metabolic byproducts excreted by ciliates likely serving as nutrients for bacterial cells. These findings suggest that conjugative transfer of mobile genetic elements within ciliate vesicles may be influenced by fluctuating conditions in WWTPs. Given the frequent interactions between bacterivorous ciliates and bacteria in WWTP influents, vesicle egestion could promote *vanA* gene transfer to related or permissive bacteria, highlighting ciliate vesicles as potential hotspots for ARG persistence in wastewater environments.

## Figures and Tables

**Table 1:** Mean vancomycin resistance gene transfer frequency between the donor and recipient *E. faecalis* strains with 10^5^ N/mL *Tetrahymena pyriformis*.

Recipient Strain	Incubation Time, h	Treatment			Control		
		Recipient Colony Count, RCC (CFU/mL)	Transconjugant Count, TC (CFU/mL) µ ± SEM, *n* = 16	Gene Transfer Frequency (TC: RCC)	Recipient Colony Count, RCC (CFU/mL)	Transconjugant Count, TC (CFU/mL) µ ± SEM, *n* = 16	Gene Transfer Frequency (TC: RCC)
ST02103^Rif^	0	1.3 × 10^9^	256 ± 14	2.0 × 10^−7^	1.3 × 10^9^	228 ± 13	1.9 × 10^−7^
	2	2.0 × 10^9^	2002 ± 49	1.0 × 10^−6^	2.6 × 10^9^	171 ± 12	6.7 × 10^−8^
	8	1.3 × 10^9^	1198 ± 28	9.6 × 10^−7^	2.2 × 10^9^	129 ± 12	6.3 × 10^−8^
	24	8.4 × 10^6^	118 ± 11	2.0 × 10^−5^	2.0 × 10^6^	37 ± 7	2.0 × 10^−5^
MW01105^Rif^	0	1.0 × 10^9^	39 ± 5	4.3 × 10^−8^	1.0 × 10^9^	38 ± 5	4.1 × 10^−8^
	2	2.7 × 10^9^	66 ± 9	2.5 × 10^−8^	3.3 × 10^9^	27 ± 5	8.4 × 10^−9^
	8	1.3 × 10^9^	10 ± 2	6.9 × 10^−9^	3.0 × 10^9^	19 ± 4	6.5 × 10^−9^
	24	4.1 × 10^6^	0	0	2.6 × 10^6^	6 ± 2	6.3 × 10^−7^

µ = mean, SEM = Standard error of the mean, *n* = number of replicates.

## Data Availability

Data will be made available upon request to the corresponding author.

## Supplementary Materials

The following supporting information can be downloaded at: https://www.mdpi.com/article/10.3390/antibiotics14050448/s1, Table S1: Data from conjugation assays with exponential-at-inception Enterococcus faecalis; Table S2: Data from conjugation assays with stationary-at-inception Enterococcus faecalis; Table S3: Data from conjugation assays with phenotypically distinct recipient *Enterococcus faecalis* strains; Table S4: Optical density (OD) readings for bacterial growth in ciliate-conditioned media; Table S5: Effect of glucose addition on conjugation assays.

## Abbreviations

AMR	Antimicrobial resistance
ANOVA	Analysis of variance
ARB	Antibiotic-resistant bacteria
ARGs	Antibiotic resistance genes
BOD	Biological oxygen demand
CFU	Colony forming unit
DNA	Deoxyribonucleic acid
MDR	Multidrug resistant
MGEs	Mobile genetic elements
PPY	Proteose peptone yeast
TC	Transconjugant count
TSA	Tryptone soya agar
TSB	Tryptone soya broth
VBNC	Viable but non-culturable
VRE	Vancomycin resistant enterococci
WWTPs	Wastewater treatment plants
